# Formulation, and Evaluation of Pentoxifylline-Loaded Poly(ε-caprolactone) Microspheres

**DOI:** 10.4103/0250-474X.42985

**Published:** 2008

**Authors:** S. Tamizharasi, J. C. Rathi, V. Rathi

**Affiliations:** Nandha College of Pharmacy, Perundurai Main Road, Erode-638 052, India; 1 Department of Pharmacy, Barakatullah Vishwavidyalaya, Bhopal-462 026, India

**Keywords:** Pentoxifylline, microspheres, poly(ε-caprolactone), biodegradable

## Abstract

Pentoxifylline-loaded poly(ε-caprolactone) microspheres were prepared by solvent evaporation technique with different drug to carrier ratio F1 (1:3), F2 (1:4), F3 (1:5) and F4 (1:6). The microspheres were characterized for particle size, scanning electron microscopy, FT-IR study, percentage yield, drug entrapment, stability studies and for *in vitro* release kinetics. The shape of microspheres was found to be spherical by SEM. The size of microspheres was found to be ranging 59.3±6.3μm to 86.22±4.23 μm. Among the four drug to carrier ratio, F3 (1:5) showed maximum percentage yield of 83.34±2.46% and F2 (1:4) showed highest drug entrapment of 76.92±3.24% w/w. It was found that there was no interaction between drug and polymer by FT-IR study. No appreciable difference was observed in the extent of degradation of product during 60 d in the microspheres, which were stored at various temperatures. In the *in vitro* release study formulation F2 (1:4) showed 90.34% drug release at 15 h and found to be sustained. The release followed Higuchi kinetics indicating diffusion controlled drug release.

The main objective of any drug therapy is to achieve a desire concentration of the drug in blood or tissue which is therapeutically effective and nontoxic for extended period of time, and this goal can be achieved by proper design of sustain release dosage regimen^1^,^2^. Microspheres have been widely accepted as a mean to achieve oral and parenteral controlled release^3^,^4^. The microspheres require a polymeric substance as a coating material or carrier^5^. A number of different substances biodegradable as well as non-biodegradable have been investigated^6^ for the preparation of microspheres. Of the various biodegradable polymers used for the development of sustained release formulations, poly(ε-caprolactone) has been reported to be advantageous since they are biocompatible^7^,^8^. Poly(ε-caprolactone) is aliphatic polyester polymer, suitable for controlled drug delivery due to a high permeability to many drugs and at the same time being free from toxicity^9^,^10^.

Pentoxifylline, a xanthine derivative, is an analogue of theophylline and inhibits phosphodiesterase. Pentoxifylline and its metabolites improve the flow properties of blood by decreasing its viscosity. In patient with chronic peripheral arterial disease, this increase blood flow to the affected microcirculation and enhance tissue oxygenation. Pentoxifylline has short half-life of 1.6 h and low oral availability (19±13%)^11^. The aim of this study was to prepare poly(ε-caprolactone) microspheres containing pentoxifylline to achieve a controlled drug release profile suitable for peroral administration.

## MATERIALS AND METHODS

Pentoxifylline was obtained as a gift sample from Shreya Health Care, Aurangabad. Poly(ε-caprolactone) was obtained from Fulka Cemika, Sigma-Aldrich Chemie, Switzerland. Dichloromethane was procured from Loba Chem. Pvt. Ltd., Mumbai. All other reagents used were of analytical grade.

### Preparation of microspheres:

Poly(ε-caprolactone) microspheres were prepared by solvent evaporation technique^12^,^13^. Drug to carrier ratio for different formulation was 1:3 (F1), 1:4 (F2), 1:5 (F3) and 1:6 (F4). Accurately weighed quantity of the poly(ε-caprolactone) was dissolved in 10 ml of dichloromethane than 200 mg of pentoxifylline was dissolved in this polymer phase. This solution was poured in 100 ml of liquid paraffin containing 1.3% Tween 80 and continuous stirred for 5 h at 1100 rpm. The microspheres were filtered and washed three times with 50 ml of n-hexane and dried at room temperature for 12 h. Microspheres dried at room temperature were then weighed and the yield of microspheres preparation was calculated using the formula^14^, Percent yield = (Amount of microspheres obtained/The theoretical amount)×100

### Evaluation of the microspheres:

Pentoxifylline was extracted from the microspheres after crushing with phosphate buffer pH 7.4 and absorbance was measured using UV/Vis spectrophotometer (Shimadzu 1601, Japan) at 274 nm. Amount of pentoxifylline in the microspheres was estimated with the help of a standard graph. Particle size analysis was carried out using optical microscopy^15^. About 200 microspheres were selected randomly and their size was determined using optical microscope fitted with a standard micrometer scale. The surface morphology and the internal textures of microsphers were observed under a scanning electron microscope^16^ (Jeol JSM-5610, Japan). FT-IR spectra of pentoxifylline, and poly(ε-caprolactone) microsphere loaded with pentoxifylline were taken to check drug polymer interaction and degradation of drug during microencapsulation.

### Stability studies:

The microspheres were placed in screw capped glass container and stored at ambient humidity conditions, at room temperatures (27±2^°^), oven temperature (40±2^°^) and in refrigerator (5-8^°^) for a period of 60 d, the microspheres were analyzed for drug content^17^.

### *In vitro* release studies:

The *in vitro* release profile of pentoxifylline from microspheres was examined in phosphate buffer pH 7.4 using the rotating paddle method (Electro Lab, Mumbai) under sink conditions^18^. Accurately weighed samples of microspheres were added to dissolution medium kept at 37±0.5^°^. At preset time intervals aliquots were withdrawn and replaced by an equal volume of dissolution medium to maintain constant volume. After suitable dilution the samples were analyzed spectrophotometrically at 274 nm^19^.

### Kinetics of drug release:

In order to understand the mechanism and kinetics of drug release, the result of the *in vitro* dissolution study of microspheres were fitted with various kinetic equations, like zero order^20^ (percentage release vs. time), first order^21^ (log percentage of drug remaining to be released vs. time) and Higuchi’s model^22^ (Percentage drug release vs. square root of time). Correlation coefficient (r^2^) values were calculated for the linear curves obtained by regression analysis of the above plots.

## RESULTS AND DISCUSSION

**Fig. 1:**
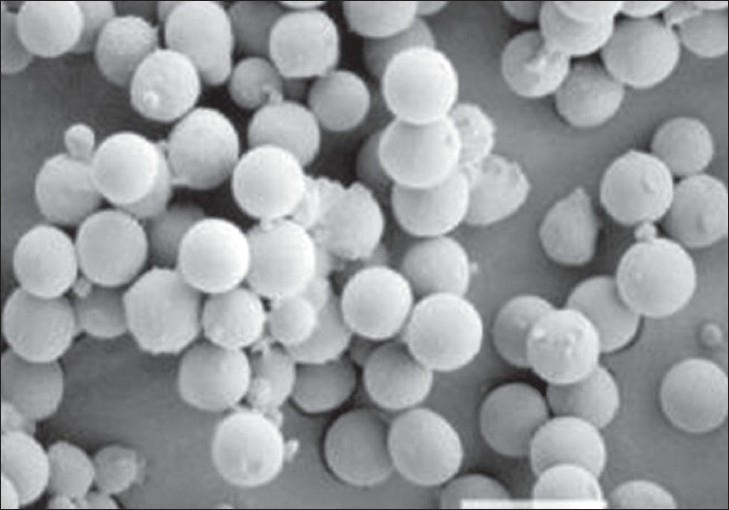
Scanning Electron Microphotograph of pentoxifylline loaded Poly(ε-caprolactone) microspheres Scanning electron microphotograph of pentoxifylline loaded Poly(ε- caprolactone) microspheres was recorded at 200 X magnification to characterize shape and surface properties of the microspheres.

Poly(ε-caprolactone) microspheres of pentoxifylline were prepared by solvent evaporation technique. Poly(ε-caprolactone) was selected as a polymer for the preparation of microspheres due to its biodegradable and biocompatible properties. The scanning electron microphotograph of microspheres is shown in fig. 1, it indicated that microspheres were spherical and discrete. The particle size was analyzed by optical microcopy. The particle size differed due to variation in the composition of the formulation. The particle size gradually increased with increasing in the proportion of poly(ε-caprolactone). The mean particle size of the microspheres is shown in Table 1. The percentage yield and entrapment efficiency were high for all the formulations and were in the range of 79.63±2.49-83.34±2.46% and 71.96±2.94-76.92±3.24% w/w, respectively, as shown in Table 1. Among the 4 drugs to carrier ratio F_3_ showed maximum percentage yield of 83.34±2.46% and F_2_ showed highest drug entrapment of 76.92±3.24% w/w.

**TABLE 1:** YIELD, DRUG ENTRAMENT AND AVERAGE PARTICLE SIZE OF PENTOXIFYLLINE LOADED POLY (ε-CAPROLACTONE) MICROSPHERES

Formulation code	Drug: polymer	Percent yield*	Drug entrapment* % w/w	Average particle size* (μm)
F1	1 : 3	79.63±2.49	73.14±2.64	59.3±6.31
F2	1 : 4	80.97±3.66	76.92±3.24	65.6±4.46
F3	1 : 5	83.34±2.46	74.69±2.38	78.52±7.41
F4	1 : 6	81.28±4.00	71.96±2.94	86.22±4.23

* Average of three preparation ± SD

**Fig. 2:**
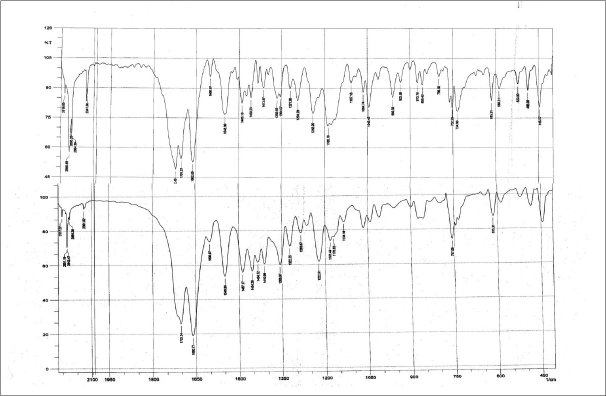
FT-IR Spectra obtained for pure pentoxifylline and pentoxifylline loaded poly (ε-caprolactone) microspheres FT-IR spectra obtained for pentoxifylline and pentoxifylline loaded poly(ε-caprolactone) microspheres were recorded to check drug-polymer interaction.

The FT-IR spectra obtained for pentoxifylline and pentoxifylline-loaded poly(ε-caprolactone) microspheres (fig. 2). The result indicated that the characteristic peaks due to pure pentoxifylline have appeared in microspheres, without any change in their position after successful encapsulation, indicating no chemical interaction between pentoxifylline and poly(ε-caprolactone) and the stability of drug during microencapsulation process. In the stability studies, no appreciable difference was observed in the extent of degradation of products during 60 d in the microspheres which were stored at various temperatures.

**Fig. 3:**
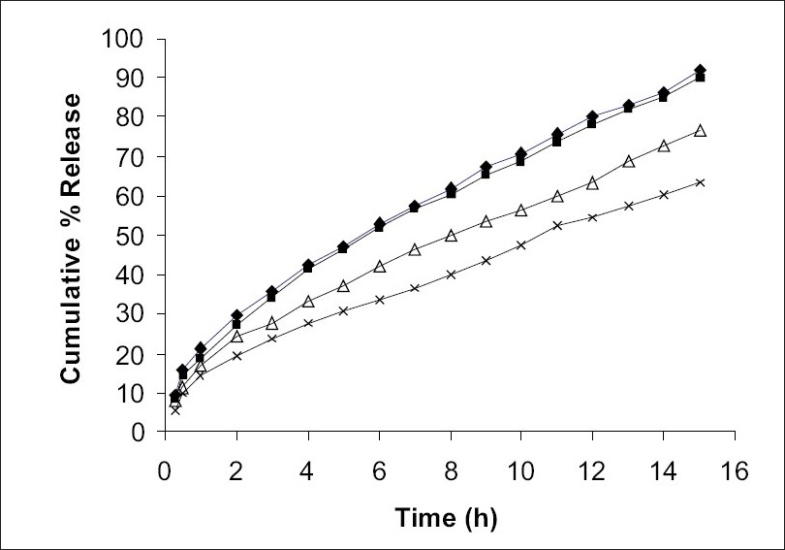
*In vitro* drug release of pentoxifylline from poly(ε caprolactone) microspheres *In vitro* dissolution profiles of pentoxifylline from poly(ε- caprolactone) microspheres formulation F1 (-♦-), F2(-▪-), F3(-Δ-) and F4(-×-) were studied in pH 7.4 phosphate buffer over a period of 15 h.

The cumulative percent release of pentoxifylline from different formulations is shown in fig. 3. Pentoxifylline release from all the formulations was slow and sustained over 15 h. The drug release rate was decreasing on increasing the polymer ratio. By the end of 15h formulation F_1_, F_2_, F_3_ and F_4_ released 92.21, 90.34, 76.65 and 63.39% of loaded drug, respectively. The polymer drug ratio 1:4 (F_2_) showed better drug entrapment and release pattern. It controlled the drug release over 15 h and was found to be the most suitable among other formulations.

**Fig. 4:**
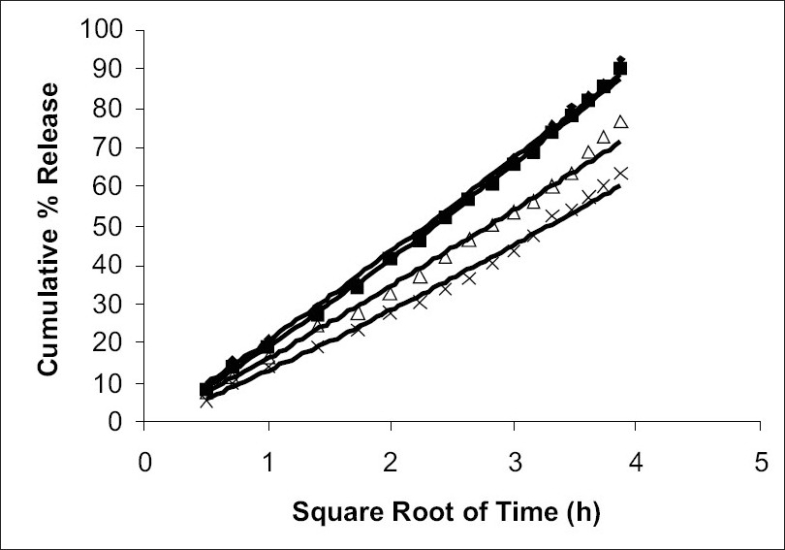
Diffusion controlled release profiles Diffusion controlled release profiles of pentoxifylline from poly(ε- caprolactone) microspheres formulation F1(-♦-), F2(-▪-), F3(-Δ-) and F4(-×-) were studied in pH 7.4 phosphate buffer.

**Fig. 5:**
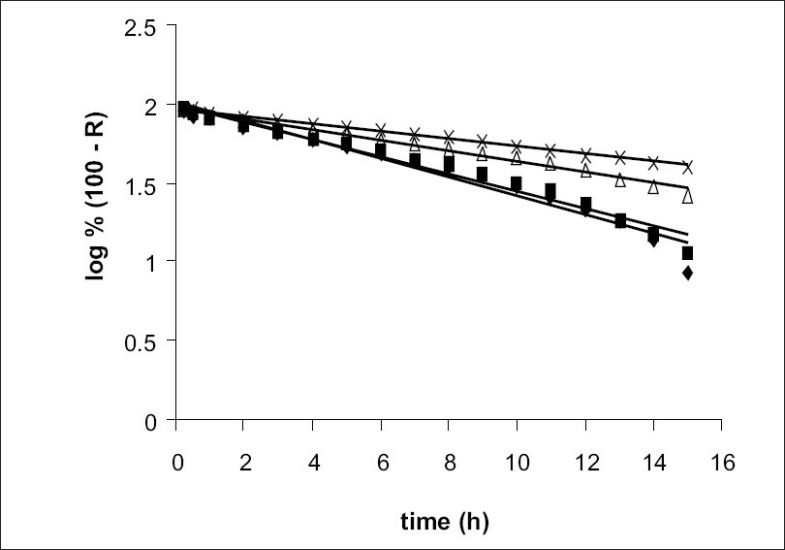
First order release profiles First order release profiles of pentoxifylline from poly(ε-caprolactone) microspheres formulation F1(-♦-), F2(-▪-), F3(-Δ-) and F4(-×-) were studied in pH 7.4 phosphate buffer.

The *in vitro* release data were applied to various kinetics models to predict the drug release mechanism and kinetics. The drug release mechanism from the microspheres was diffusion controlled as plots of the amount released versus square root of time (fig. 4) was found to be linear. The correlation coefficient (r^2^ ) was in the range of 0.978-0.987 for various formulations as shown in Table 2. When log percentage of drug remaining to be released vs. time was plotted in accordance with first order equation, straight lines were obtained (r^2^>0.95) indicated that drug release followed first order kinetics (fig. 5).

**TABLE 2:** VALUE OF R^2^ FROM RELEASE DATA OF VARIOUS FORMULATIONS FOR DIFFERENT MODLES OF MECHANISMS OF DRUG RELEASE

Model	F1	F2	F3	F4
Zero order	0.853	0.865	0.883	0.877
First order	0.994	0.983	0.965	0.954
Higuchi	0.987	0.985	0.978	0.979

The values listed are the values of coefficient correlation (r^2^) obtained from release data of various formulations for different models of mechanism of drug release.

In present study, an attempt was made to prepare pentoxifylline microspheres using a biodegradable, biocompatible carrier, poly(ε-caprolactone) by solvent evaporation technique. The method was found to be simple and reproducible. It may be concluded from the result obtained from evaluation and performance study of microspheres that system may be useful to achieve a controlled drug release profile suitable for peroral administration and may help to reduce the dose of drug, dosing frequency and improve patient compliance.
